# Evaluation of ^18^F-IAM6067 as a sigma-1 receptor PET tracer for neurodegeneration *in vivo* in rodents and in human tissue

**DOI:** 10.7150/thno.47585

**Published:** 2020-06-29

**Authors:** François-Xavier Lepelletier, Matthias Vandesquille, Marie-Claude Asselin, Christian Prenant, Andrew C Robinson, David M A Mann, Michael Green, Elizabeth Barnett, Samuel D Banister, Marco Mottinelli, Christophe Mesangeau, Christopher R McCurdy, Inga B Fricke, Andreas H. Jacobs, Michael Kassiou, Hervé Boutin

**Affiliations:** 1 Faculty of Biology, Medicine and Health, School of Biological Sciences, Division of Neuroscience and Experimental Psychology, University of Manchester, Manchester, United Kingdom.; 2 Wolfson Molecular Imaging Centre, University of Manchester, Manchester, United Kingdom.; 3 Faculty of Biology, Medicine and Health, School of Health Sciences, Division of Informatics, Imaging and Data Sciences, University of Manchester, Manchester, United Kingdom.; 4 Salford Royal NHS Foundation Trust, Department of Clinical & Cognitive Neurosciences, Clinical Sciences Building, Salford, United Kingdom.; 5 School of Chemistry, The University of Sydney, Sydney, Australia.; 6 Department of Medicinal Chemistry, College of Pharmacy, University of Florida, Gainesville, FL 32610, USA.; 7 Department of BioMolecular Sciences, School of Pharmacy, University of Mississippi, University, MS 38677, USA.; 8 UF Translational Drug Development Core, University of Florida, Gainesville, FL 32610, USA.; 9 European Institute for Molecular Imaging (EIMI), Westfälische Wilhelms-Universität (WWU), Münster, Germany.; 10 Department of Geriatrics and Neurology, Johanniter Hospital, Bonn, Germany.

**Keywords:** Sigma 1 receptor, PET radiotracer, Parkinson's disease, Alzheimer's disease, animal model

## Abstract

The sigma 1 receptor (S1R) is widely expressed in the CNS and is mainly located on the endoplasmic reticulum. The S1R is involved in the regulation of many neurotransmission systems and, indirectly, in neurodegenerative diseases. The S1R may therefore represent an interesting neuronal biomarker in neurodegenerative diseases such as Parkinson's (PD) or Alzheimer's diseases (AD). Here we present the characterisation of the S1R-specific ^18^F-labelled tracer ^18^F-IAM6067 in two animal models and in human brain tissue.

**Methods:** Wistar rats were used for PET-CT imaging (60 min dynamic acquisition) and metabolite analysis (1, 2, 5, 10, 20, 60 min post-injection). To verify *in vivo* selectivity, haloperidol, BD1047 (S1R ligand), CM398 (S2R ligand) and SB206553 (5HT_2B/C_ antagonist) were administrated for pre-saturation studies. Excitotoxic lesions induced by intra-striatal injection of AMPA were also imaged by ^18^F-IAM6067 PET-CT to test the sensitivity of the methods in a well-established model of neuronal loss. Tracer brain uptake was also verified by autoradiography in rats and in a mouse model of PD (intrastriatal 6-hydroxydopamine (6-OHDA) unilateral lesion). Finally, human cortical binding was investigated by autoradiography in three groups of subjects (control subjects with Braak ≤2, and AD patients, Braak >2 & ≤4 and Braak >4 stages).

**Results:** We demonstrate that despite rapid peripheral metabolism of ^18^F-IAM6067, radiolabelled metabolites were hardly detected in brain samples. Brain uptake of ^18^F-IAM6067 showed differences in S1R anatomical distribution, namely from high to low uptake: pons-raphe, thalamus medio-dorsal, *substantia nigra*, hypothalamus, cerebellum, cortical areas and striatum. Pre-saturation studies showed 79-90% blockade of the binding in all areas of the brain indicated above except with the 5HT_2B/C_ antagonist SB206553 and S2R ligand CM398 which induced no significant blockade, indicating good specificity of ^18^F-IAM6067 for S1Rs. No difference between ipsi- and contralateral sides of the brain in the mouse model of PD was detected. AMPA lesion induced a significant 69% decrease in ^18^F-IAM6067 uptake in the globus pallidus matching the neuronal loss as measured by NeuN, but only a trend to decrease (-16%) in the caudate putamen despite a significant 91% decrease in neuronal count. Moreover, no difference in the human cortical binding was shown between AD groups and controls.

**Conclusion:** This work shows that ^18^F-IAM6067 is a specific and selective S1R radiotracer. The absence or small changes in S1R detected here in animal models and human tissue warrants further investigations and suggests that S1R might not be the anticipated ideal biomarker for neuronal loss in neurodegenerative diseases such as AD and PD.

## Introduction

Sigma receptors have drawn particular interest over the years as potential biomarkers of neurodegeneration 1-4. Pharmacological studies have reported the evidence of at least two subtypes, i.e. sigma 1 (σ1) and sigma 2 (σ2) receptors 5, 6. The σ1 receptor (S1R) is a highly conserved 25 kD molecular chaperone protein with 223 amino acids 6, 7. *In vitro* crystallography revealed that the S1R is found in a homotrimeric form with 1 transmembrane region per protomer 8, although *in vivo* studies and sequence-based models support 2 transmembrane domains, which according to Penke *et al.* suggest a “potentially amorphous intrinsically disordered *in vivo* structure” of S1R 6. The S1R is expressed mostly in the brain, in neurons, oligodendrocytes and ependymal cells, but also in peripheral organs and tissue (for review see 6). At a cellular level, S1R is mainly located at the endoplasmic reticulum (ER), in close contact with mitochondria at the mitochondria-associated ER membrane (MAM) 6, 9, although S1R can easily translocate to the plasma membrane, the nuclear envelope or interact with cytosolic proteins with consequences for regulation of Ca^2+^ signalling associated cell survival (for review see 6 & 9).

The S1R is involved in the regulation of many neurotransmission systems such as cholinergic, noradrenergic and dopaminergic systems 10, although its exact role is still poorly understood. This neuro-modulatory role means that S1R is indirectly involved in various neuropathology, including schizophrenia, depression, stroke, Parkinson's disease (PD) and Alzheimer's disease (AD) 1, 11, 12. Some studies have shown a link between S1R dysregulation and amyotrophic lateral sclerosis, AD or PD progression 3, 13-16. Other studies have demonstrated that there is a reduction in the anterior putamen S1R density in PD patients 17 and also a reduction in cortical and cerebellar S1R density in brains of AD patients 18 and in AD *post-mortem* tissues 19, 20. S1R expression has therefore been envisaged as a potential biomarker for neuronal function in health and diseases.

Previously developed S1R PET radiotracers had limited and variable affinity and/or selectivity towards S1R, with most of them displaying some capacity to bind σ2 receptors and/or other neuro-receptors (for review see 21, 22). Amongst the S1R radiotracers, ^11^C-SA4503 (K_i_ = 4.4 nM, K_i σ2/σ1_ = 55) 23 has been the most used 18, 24, 25 with other tracers presenting various levels of selectivity (for review see 22). Amongst all the [^18^F]-labelled S1R radiotracers, ^18^F-FTC-146 (K_i_ = 2.5 pM, K_i σ2/σ1_ = 144,800) 26, ^18^F-fluspidine (K_i_ = 0.59 nM, K_i σ2/σ1_ = 1330) 27 and ^18^F-IAM6067 (K_i_ = 2.6 nM, K_i σ2/σ1_ = 187), presented here, are the most selective for S1R *vs* S2R 22, 28. Selectivity of ^18^F-IAM6067 for S1R against a broad range of receptors and transporters has been previously reported by Moussa *et al.* (see supplementary information in 29 where ^18^F-IAM6067 is compound #30). Although affinity and selectivity are essential criterion, *in vivo* pharmacokinetics is an equally important parameter as recent studies have shown that ^18^F-FTC-146 had unfavourably slow pharmacokinetic properties for human use 30, 31. Noteworthily, it was shown in non-human primate that despite having lower affinity (S)-^18^F-fluspidine (K_i_ = 2.30 nM) displayed more favourable pharmacokinetics for human use than (R)-^18^F-fluspidine (K_i_ = 0.52 nM) 32. Hence, taking into account all these considerations, to date (S)-^18^F-fluspidine appears to be the most suitable candidate for S1R PET imaging in human.

**Figure 1:**
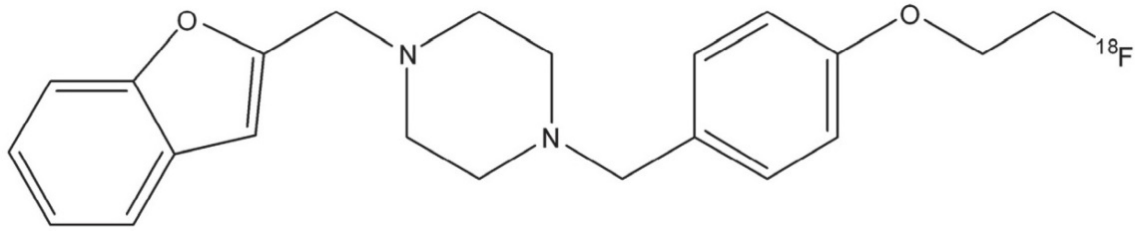
Chemical structure of *N*-(2-Benzofuranylmethyl)-*N*'-[4-(2-fluoroethoxy)benzyl]piperazine (^18^F-IAM6067).

In this study, we present the validation of the S1R specific ^18^F-labelled tracer ^18^F-IAM6067 28 (Figure 1). The specificity towards S1R was initially evaluated in a *Papio hamadryas* baboon pre-treated with haloperidol (1 mg/kg, i.v.) which resulted in a 80% displacement in specific receptor binding in all areas of the brain 28. Therefore, the aims of this study were to fully evaluate the *in vivo* brain uptake of ^18^F-IAM6067 using *in vivo* PET imaging; we also tested its specificity and selectivity for S1R, S2R and 5HT_2B_ as Moussa *et al.* 29 reported some affinity for those receptors (K_i σ2/σ1_ = 187, K_i 5HT2B/σ1_ = 37). *In vivo* data were confirmed by *in vitro* autoradiography. Furthermore to investigate the potential of S1R as neuronal biomarker, we studied ^18^F-IAM6067 binding *in vivo* and by autoradiography in a model of AMPA lesion in Wistar rats, by autoradiography in a mouse model of PD, and in AD human tissue as classified by Braak staging 33.

## Materials and Methods

### Radiosynthesis

*N*-(2-Benzofuranylmethyl)-*N'*-[4-(2-fluoroethox)benzyl]piperazine (IAM6067) was labelled with [^18^F]. The radiosynthesis was previously described by Moussa and colleagues 28.

### Animals

Forty male Wistar rats (Charles River, Margate, UK) were used for in this study. Animals weighed 378 ± 121 g at the time of experimentation and were kept under a 12-hour light/dark cycle under steady temperature and humidity with access to food and water *ad libitum*. All procedures were carried out in accordance with the Animals (Scientific Procedures) Act 1986, and the specific project licence was approved by the UK Home Office.

### Metabolite analysis in plasma and brain

Twenty-four rats were anesthetized using isoflurane (induction 4-5%, 2-2.5% thereafter in 70%/30% NO_2_/O_2_ mixture at 1 L/min) and catheterized (Jelco® I.V. catheter 24G) in the tail vein for ^18^F-IAM6067 administration (32-123 MBq). Blood samples were collected at 1, 2, 5 min post-injection and at 10, 20, 40 or 60 min post-injection when the animals were sacrificed. Seven animals used for metabolites analysis were also scanned for 60 min. Plasma was isolated by centrifugation (3 min, 3000 × g at 4 °C). Two hundred µL of CH_3_CN was added to 200 µL of serum for protein precipitation and after centrifugation (3 min, 3000 × g at 4 °C), 100 µL of supernatant was injected into the HPLC for quantification (HPLC equipment: Shimadzu prominence with a UV detector and bespoke Scintillation detector; column: ACE 3 C18 HL, [Hichrom], 150 × 4.6 mm; porosity: 3 μm; solvent A: 0.1 M ammonium acetate; solvent B: CH_3_CN, isocratic elution: 50:50 (A/B) in 15 min; flow rate: 1 mL/min; room temperature; absorbance detection at λ = 254 nm). Animal brains were removed and then homogenised by ultra-Turrax® in 2 mL of ice-cold acetonitrile. After centrifugation (3 min, 9000 × g at 4 °C), the supernatant was separated from the pellet. One hundred microliters of brain supernatant were injected on the HPLC column (same conditions as above). Control samples were spiked with 15 µL of stable standard. The parent radiotracer eluted at 11 minutes.

### AMPA lesion in Wistar rats

Three rats were anesthetized using isoflurane (induction 4-5%, 2-2.5% thereafter in 70%/30% NO_2_/O_2_ mixture) and stereotaxically injected with 7.5 nmol of α-amino-3-hydroxy-5-methyl-4-isoxazolepropionic acid (AMPA, 7.5 mM in PBS buffer, Tocris Bioscience, UK) through the use of a 2 µL microsyringe (Hamilton Neuros syringe Model 7002KH point style 3) and micropump (injection rate: 0.5 µL/min, UltraMicroPump II and Micro4 Controller, WPI Inc., USA) in the right striatum (Bregma +0.7 mm, from sagittal suture: 3 mm, depth from brain surface: 5.5 mm). Animals were maintained normothermic (body temperature: 37.0 ± 0.4 °C) during the surgery through the use of a heating blanket (Homeothermic Blanket Control Unit, Harvard Apparatus Limited®, Edenbridge, Kent, UK). Twenty-four hours later, the rats were anesthetized again as described above and scanned with a 3T cryogen-free MRS 3000 scanner with a quadrature, rat head coil (MR Solutions, Guildford, UK) 34, 35 to assess oedema (i.e. lesion size and location) immediately before the PET scan. Parameters for the T2-weighted RARE sequence were: repetition time = 4800 ms, base echo time = 17 ms, effective echo time = 68 ms, number of echoes = 8, bandwidth = 20 kHz, number of samples = 256, number of views = 240, number of averages = 1 with 25 slices of 1.06 mm, giving a final voxel size of 0.136 × 0.136 × 1.06 mm.

### Animal PET and data acquisition

Sixty minutes PET scans were performed on 23 animals on a Siemens Inveon® PET/CT scanner (Injected dose: 38±11MBq). Eight of them were used for basal uptake. To assess the *in vivo* specificity of ^18^F-IAM6067, 2 of those eight rats were also used for the pre-saturation protocol (BD1047) on a different day. Nine rats were scanned for pre-saturation studies with haloperidol (n = 3 scans) and/or BD1047 (n=4 scans) and/or SB206553 (n = 5 scans) on different days. An additional three rats were scanned with ^18^F-IAM6067 alone and 3 days later with ^18^F-IAM6067 after pre-treatment with the S2R antagonist CM398 36. These pre-saturation studies were conducted by injecting 1mg/kg of haloperidol (D2/σ receptor antagonist; Tocris Bioscience, UK; n = 3) 37, 38, BD1047 (S1R antagonist; Tocris Bioscience, UK; n = 4) 39, SB206553 (5-HT_2B_/5-HT_2C_ receptor antagonist; Tocris Bioscience, UK; n = 5) or CM398 (S2R antagonist, kindly provided by Dr McCurdy; n = 3) i.v. 20-30 min before scan start. Animals scanned on multiple occasions (with/without blocking or different blocking agent) were always scanned at least 24 h apart. Three additional rats received intra-striatal AMPA and were scanned with ^18^F-IAM6067 24 h after lesion. Each rat was anesthetized using isoflurane (induction 4-5%, 2-2.5%) and catheterized in the tail vein for blocking agent and ^18^F-IAM6067 injection. Animal respiration and temperature were controlled using a pressure-sensitive pad and rectal probe (BioVet, m2m Imaging Corp., Cleveland, OH, USA) and body temperature was maintained at 37 ± 0.7 °C via the interface managed by the BioVet system. PET-CT was performed as previously described 40. Briefly, a CT scan was performed prior to the PET acquisition for attenuation correction. The list mode emission data were histogrammed into 3D sinograms of 35 dynamic frames, normalized, corrected for dead-time, attenuation, scatter and radioactive decay, and reconstructed using OSEM3D (16 subsets and 4 iterations) into images of dimensions 128 × 128 × 159 voxels of volume 0.776 × 0.776 × 0.796 mm^3^.

### PET image analysis

PET-CT images were co-registered with the rat MRI template and its associated intracranial mask as published by Schwarz et al. 41 and generously provided by GSK. To co-register the MRI template and PET-CT images, individual brain masks were delineated on each CT image and co-registered with the MRI brain mask using rigid registration with mutual information. Image analysis and quantification was performed using the BrainVisa/Anatomist software (http://www.brainvisa.info) using the geometric transfer matrix (GTM) and ROIopt methods for partial volume correction both described and used previously 42-46. Modified larger ROIs were drawn on the MRI anatomical template 41 for ^18^F-IAM6067 quantification. PET data were expressed as SUV.

### Parkinson's disease mouse model

C57Bl6 mice were bred in the animal facility of the European Institute for Molecular Imaging (University of Muenster). Animal experiments were performed in accordance with the German laws for animal protection and were approved by the local bureau for animal care (LANUV, Landesamt für Natur, Umwelt und Verbraucherschutz Nordrhein-Westfalen). For stereotactic injections, mice (n = 6) were anaesthetised (ketamine/xylazine i.p.) and placed into a stereotactic frame. A small skin incision followed by craniotomy was performed. The needle was placed in position (coordinates: AP: +0.5 mm; ML: -2.0 mm; DV: -3.0 mm) and after 1 min, 2 µL of 5 mg/mL 6-hydroxydopamine (6-OHDA) in 0.9% NaCl and 0.02% ascorbic acid were injected into the left striatum using a 5 µL Hamilton 7005KH syringe. The syringe was kept in place for 5 min in order to allow the solution to diffuse into the surrounding tissue and was then retracted slowly.

### Human tissue

*Post-mortem* brain tissues of 23 cases (8 men and 15 women) were obtained from the Manchester Brain Bank through appropriate consenting procedures for collection and use of the human brain tissues. Pathological diagnoses were made by an experienced neuropathologist (DM) as previously described 47, 48 and were in accordance with recent National Institute on Ageing - Alzheimer's Association guidelines for the neuropathological assessment of AD 49. By such classification, only cases with Braak stages 5 and 6 were classed as definite AD. Cases with Braak Stages >2 & ≤4 were not diagnosed as definite AD, but these could be considered as cases of possible (incipient) AD, thereby representing cases with early changes of AD. Amyloid plaque load *per se* was not employed for classification of AD, although it is acknowledged that neuritic plaque scores do form the basis of CERAD classification. Clearly, in terms of amyloid plaques alone, there was overlap in amyloid load between cases at Braak stage >2 & ≤4 and those at Braak stages 5 and 6. Subjects were therefore split in three groups based on their Braak stages 33: controls (Braak ≤2, 8 subjects), subclinical AD (Braak >2 & ≤4, 8 subjects) and AD (Braak >4, 7 subjects). Details of each case are described in Table 1. Hence, brains in the first group were considered as a (normal) control group (Braak ≤2). These were obtained from aged patients with no history of neurological disease and no pathological evidence of AD or other degenerative brain disease. The second group (Braak >2 & ≤4) consisted of patients with mild to moderate AD-type pathology, whilst the last group (Braak >4) all showed abundant plaques and tangles throughout all cortical regions and met pathological criteria for established AD 49, 50. The average *post-mortem* delay was also similar for all groups (mean ± SD [min.-max.]: 34.9 ± 11.3 12-46; 35.8 ± 9.9 25-48 and 41.5 ± 14.2 24-64 hours for controls, subclinical AD and AD groups, respectively). Subsequently to the autoradiography experiments, measurements from frontal cortex of case #3 were not included as the sections had been damaged during the experiments (Table 1).

### Immunohistochemistry

Two weeks after 6-OHDA lesion, animals were sacrificed by cervical dislocation and brains were removed and frozen in -40°C isopentane. Cryosections of 20 µm thickness were cut every 500µm, resulting in 16 levels per brain. In order to assess the effect of the 6-OHDA on the striatal dopaminergic neurons of the C57Bl6 mice, immunohistochemistry for tyrosine-hydroxylase was performed using the following protocol: brains were post-fixed with 4% PFA in PBS for 15 min at 4 °C, then washed with PBS (3 × 5min). For antigen retrieval, sections were boiled for 25 min in citrate buffer (pH 6), left in the buffer to cool down for 20 min, and washed with PBS (3 × 5 min). Blocking was performed with Peroxidase-blocking solution (S2023, Dako, Hamburg, Germany) for 10 min followed by a PBS washing step (3 × 5 min). After incubation with primary antibody (chicken anti-tyrosine-hydroxylase, Abcam ab76442, 1:800, Abcam, Cambridge, UK) over night at 4 °C in antibody diluent (S3022, Dako), sections were washed (PBS, 3 × 5 min), incubated with biotinylated secondary antibody (DSB-XTM Biotin Goat Anti-Chicken IgG 1:800, Life Technologies, Carlsbad, CA, USA) in antibody diluent for 60min, washed (PBS, 3 × 5 min) and incubated with HRP Streptavidin conjugate (1:600 in PBS, P0397, Dako) for 45min. After washing (PBS, 3x5min), sections were placed in 2% 3,3'-Diaminobenzidine (DAB), 0.0012% H_2_O_2_ until reaching a good staining intensity. Sections were dehydrated in 70 %, 80 %, 96% ethanol, 100% isopropanol and xylene (each twice) and mounted in Entellan (Merck Millipore, Burlington, MA, USA).

Images were collected on a Nikon ECLIPSE Ni-E upright microscope using a 10×/0.30 or 20×/0.50 UPlanFLN objectives operated by the NIS Elements AR software (Nikon Instruments Inc., Tokio, Japan).

Immunohistochemistry was also carried out in rats that received intrastriatal AMPA to visualise NeuN (neuronal marker). Animals were culled by isoflurane overdose confirmed by cervical dislocation. The brains were collected, snap frozen using isopentane on dry ice and stored at -80 °C. Coronal brain sections (20 µm thick) were taken using a cryostat (Leica CM3050s, Leica Biosystems Nussloch GmbH, Germany) and stored at -80 °C. Sections were allowed to defrost and dry at room temperature for 20min and then fixed with 4% paraformaldehyde for 10 min before being washed (6 × 5 min) in phosphate buffered saline (PBS) and incubated for 30 min in 2% normal donkey serum and 0.1% Triton X-100 in PBS to permeabilise and block non-specific binding. Primary antibody incubation was carried out overnight at 4 °C with rabbit anti-NeuN (Abcam, ab177487, 1:500). Following incubation in primary antibody, PBS washes were repeated (3 × 10min) and incubation with the secondary antibody (Alexa Fluor 594nm Donkey anti-rabbit IgG 1:500) was carried out. Images of the lesioned striatum were collected on an Olympus BX51 upright microscope using a 10×/0.30 UPlanFLN objectives and captured using a Retiga 6000 Color and R6 camera through QCapture Pro 7 and Ocular Software (QImaging Inc.). Specific band pass filter sets were used to prevent bleed through from one channel to the next. NeuN staining was quantified using ImageJ (1.52n) 51-53 using 5 snapshots for the caudate putamen, 3 snapshots for the *globus pallidus* and 2 snapshots for the *substantia nigra* for each side of the brain (ipsi- and contralateral). Both the total number of neurons averaged over the snapshots and the percentage of stained areas were measured.

### Autoradiography

^18^F-IAM6067 autoradiography was performed using 20µm fresh frozen brain sections from 6 naïve and 3 AMPA-injected Wistar rats, 6 C57Bl6 mice and also from the 23 human cases. Sections of frontal (Brodmann areas 8/9) and temporal cortex (Brodmann areas 21/22) were cut from frozen human tissue blocks. Human and rodents sections were mounted on to glass slides (SuperFrost Plus Gold and SuperFrost Plus slides, Thermo Fisher Scientific Inc., MA, USA for human and animal tissues, respectively). For all steps described below, Tris buffer (Trizma pre-set crystals (Sigma, UK), 50 mM, with 120 mM NaCl, with pH adjusted to 7.4 at 4 °C or room temperature) was used. Slides were allowed to defrost then pre-incubated in Tris Buffer at 4 °C for 5min. Slides were then incubated with ^18^F-IAM6067 (2 nM) in Tris Buffer at room temperature for 60 min to determine total binding. Adjacent sections were incubated with the same solution containing an excess of unlabelled IAM6067 (2 µM) to assess non-specific binding. Next the slides were washed 2 × 2 min in Tris buffer at 4 °C, followed by a quick rinsing in distilled water at 4 °C. Slides were then dried promptly before exposition on Phosphor-Imager plates overnight. Autoradiographs were analysed using AIDA software (Raytest GmbH, Germany). Regions of interest (ROI) were manually drawn (amygdala, striatum, cingulate cortex, sensorimotor cortex (SS), hippocampus, hypothalamus, pons, thalamus medio-dorsal (MD), region containing the ventral tegmental area and the substantia nigra (VTA/SN) and the cerebellum for the naive rat tissue; the ipsi- and contralateral striatum (the ipsilateral striatum includes the lesioned part of both the caudate-putamen and *globus pallidus* pooled together notably because accurate anatomical delineation of both brain regions on the autoradiography was difficult) and cortex for the AMPA-injected rats; the ipsi- and contralateral parts of the cingulate cortex, striatum, CM thalamus and VTA/SN for the mouse tissue; the grey and white matter for the human tissue). Specific binding (total binding minus non-specific binding) in each ROI is expressed as intensity of the photostimulated luminescence (PSL) per pixel.

### Statistical analysis

All data are expressed as mean ± standard deviation (SD). Statistical significance was accepted at P<0.05 level. Statistical tests were performed using GraphPad Prism 8.4 for Windows (GraphPad Software, CA, USA, www.graphpad.com) and P values adjusted for multiple comparisons are reported for Sidak's post-hoc tests.

### PET data

For uptake of ^18^F-IAM6067 without and with blocking compounds, values were analysed using 2 way ANOVA (ROI as repeated factor and treatment; i.e. baseline *vs* blocking), the scans with haloperidol, BD1047 or SB206553 pre-treatment were compared to baseline scans. As the experiments with CM398 were performed later, scans without and with pre-treatment with CM398 were performed in the same animals and therefore were analysed using 2 way ANOVA with repeated measures for ROI and treatment. ANOVA were followed by a Sidak's post-hoc test. For comparisons between baseline *vs* haloperidol, BD1047 and SB206553 pre-treatments and baseline *vs* CM398, and between ROIs, considering the large number of comparisons, the more conservative option of one family for all comparisons was applied for the Sidak's post-hoc test. For the AMPA experiment, comparison of ^18^F-IAM6067 uptakes by PET in ipsi- *vs* contralateral *vs* lesion in the caudate-putamen and *globus pallidus* (as delineated on the T2 MRI) ROIs and comparisons all ROIs of control (no lesion) *vs* AMPA-injected animals were performed using a 2 way ANOVA followed by a Sidak's post-hoc test.

### Autoradiography data

Specific binding in the ipsi- and contralateral striatum in the rat AMPA model was analysed with a paired *t*-test.

Specific binding between the ipsi- and contralateral parts of each region in the PD mouse model ^18^F-IAM6067 autoradiography was analysed using a 2 way ANOVA (both ROI and side, ipsi- *vs* contralateral, as repeated factors). The 2 way ANOVA only returned an effect of ROI (p=0.003) but no significant differences between ipsi- and contralateral side or interaction (p=0.84 and p=065 respectively), therefore a Sidak's post-hoc test comparing ROIs only was performed.

Specific binding for human autoradiography across the three groups in both frontal and temporal cortex and for grey and white matter was analysed by mixed-model fitting (brain structures as repeated factor). As this analysis only returned an effect for the brain structures factor and not for the Braak stage, a Sidak's post-hoc test comparing brain regions only was performed.

### Immunohistochemistry data

For NeuN staining analysis and ^18^F-IAM6067 autoradiography in the AMPA model, percentage stained area, cell counts and ^18^F-IAM6067 were analysed using paired *t*-tests (ipsi- *vs* contralateral) for the caudate-putamen, *globus pallidus* and* substantia nigra*.

## Results

### Radiosynthesis

On average, about 2.96 GBq (range 0.53-11.3 GBq) of ^18^F-IAM6067 (>98.5% radiochemically pure) were routinely obtained within 65 min of radiosynthesis (including high-performance liquid chromatography (HPLC) purification and formulation), with molar activities ranging from 55 to 345 GBq/µmol.

### Metabolites analysis

Metabolites analysis revealed 3 fractions of the radiotracer with retention times at 1.4 and 1.8 min for metabolites and between 10.9 and 11.1 min for the parent fraction (in the plasma and the brain, respectively) (Table 2). Moreover, radiolabelled metabolites analysis showed rapid metabolism of ^18^F-IAM6067 (only 10.1 ± 8.6% of intact tracer in plasma at 10 min post-injection) (Table 2). On contrary, more than 91% of the intact ^18^F-IAM6067 was found in the brain at 10, 20 and 60 min post-injection. Metabolites represented only a minor fraction of radioactivity found in the brain (undetectable at 1, 2 and 5 min post-injection and less than 10% at 10, 20 and 60 min post-injection) (Table 2). The retention times of these 2 metabolites (1.4 and 1.8min, Table 2) suggest that they were more hydrophilic than the parent compound.

### Tracer brain uptake and distribution

**Figure 2:**
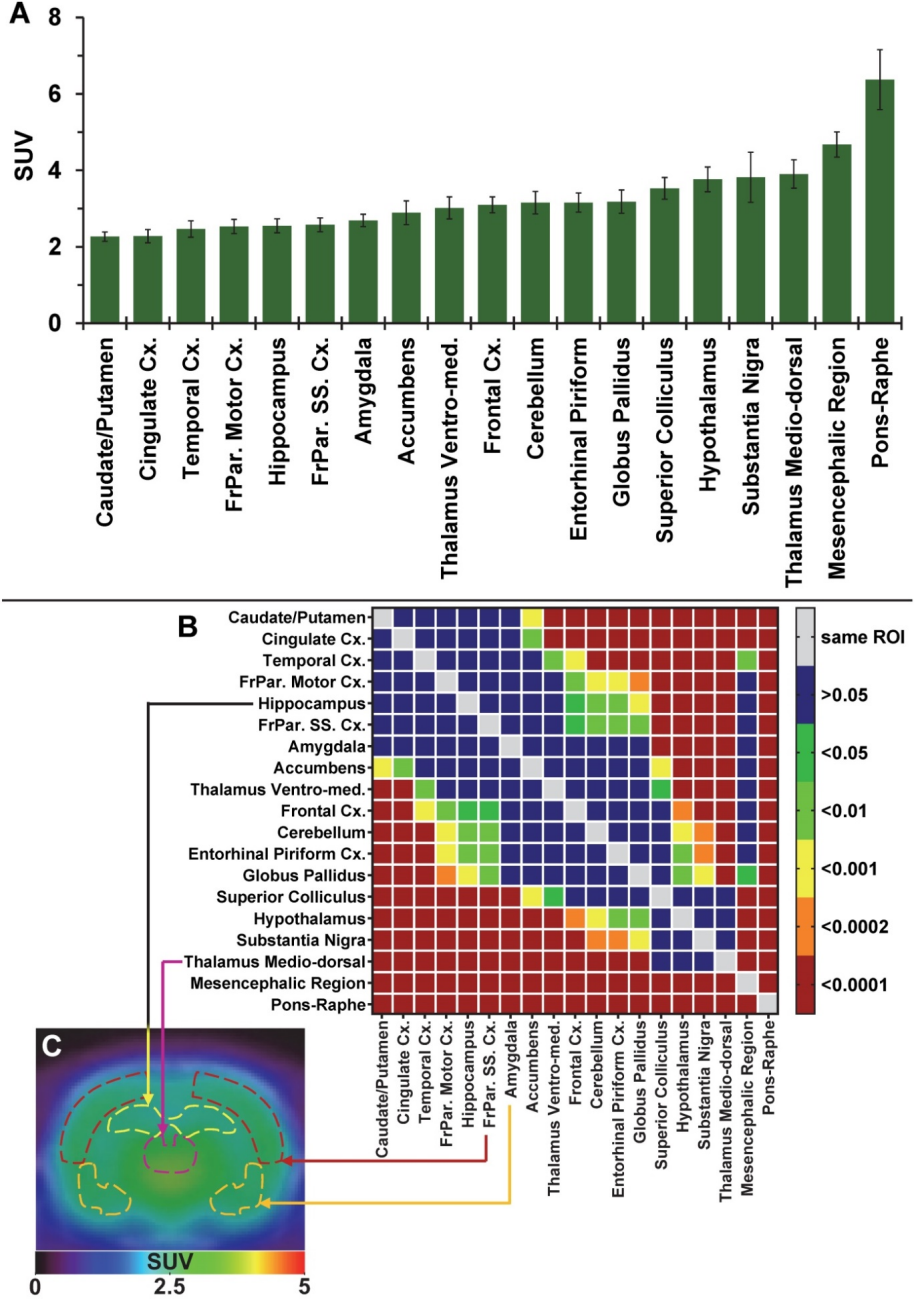
(**A**) Average uptake (from sum-image 20-60min post-injection) of ^18^F-IAM6067 in different brain regions in rats (n=8, data are expressed as SUV mean ± SD). (**B**) Heat map of the adjusted P values (Sidak's post-hoc test) showing all the comparisons between the various brain regions for ^18^F-IAM6067 uptake. Non-significant differences are shown in blue. (**C**) PET sum-image (20-60min) co-registered with CT showing ^18^F-IAM6067 uptake with hippocampus, thalamus medio-dorsal, frontoparietal somatosensory cortex and amygdala highlighted by dotted lines.

**Figure 3:**
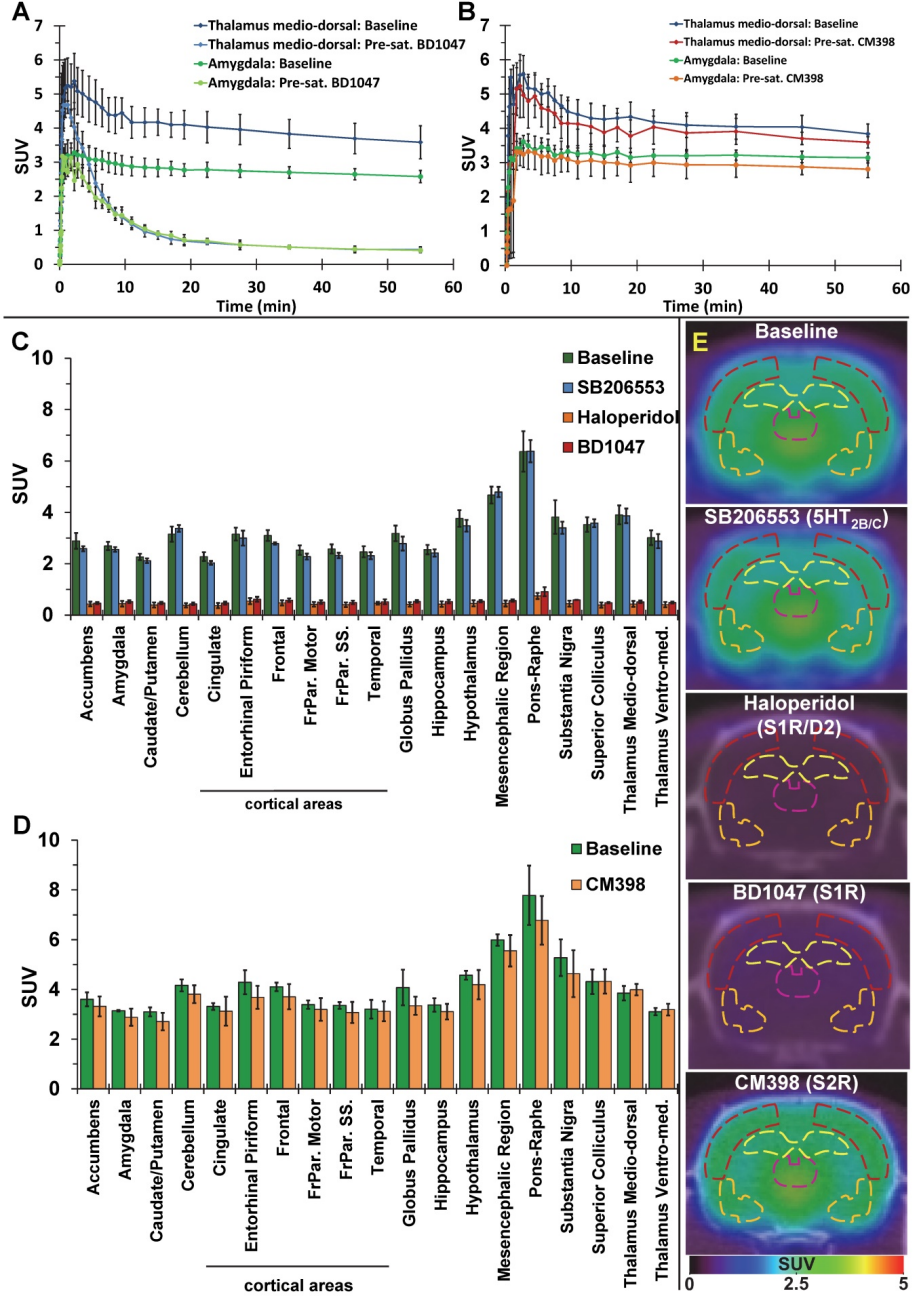
Pharmacokinetic time-activity curves in rat thalamus medio-dorsal and amygdala showing ^18^F-IAM6067 uptake at baseline (n=8) and after pre-saturation with S1R antagonist BD1047 (n=4) (**A**) or at baseline (n=3) and after pre-saturation with S2R antagonist CM398 (n=5) (**B**). (**C**) Comparison of average uptake (from sum-image 20-60min post-injection) of ^18^F-IAM6067 between baseline (no pre-treatment, n=8, green bars) and SB206553 (n=5, blue bars), haloperidol (n=3, orange bars) or BD1047 (n=4, red bars) pre-saturations and (**D**) between baseline and CM398 pre-treatment (n=3) in different brain regions (Data are expressed SUV mean ± SD). (**E**) Co-registered PET-CT images showing ^18^F-IAM6067 uptake at baseline and with pre-saturation with haloperidol, SB206553, BD1047 or CM398 (sum-image 20-60min after injection of the radiotracer); outline of the frontoparietal somatosensory cortex (dark red), hippocampus (yellow), thalamus medio-dorsal (purple) and amygdala (orange) regions of interest are shown.

**Figure 4:**
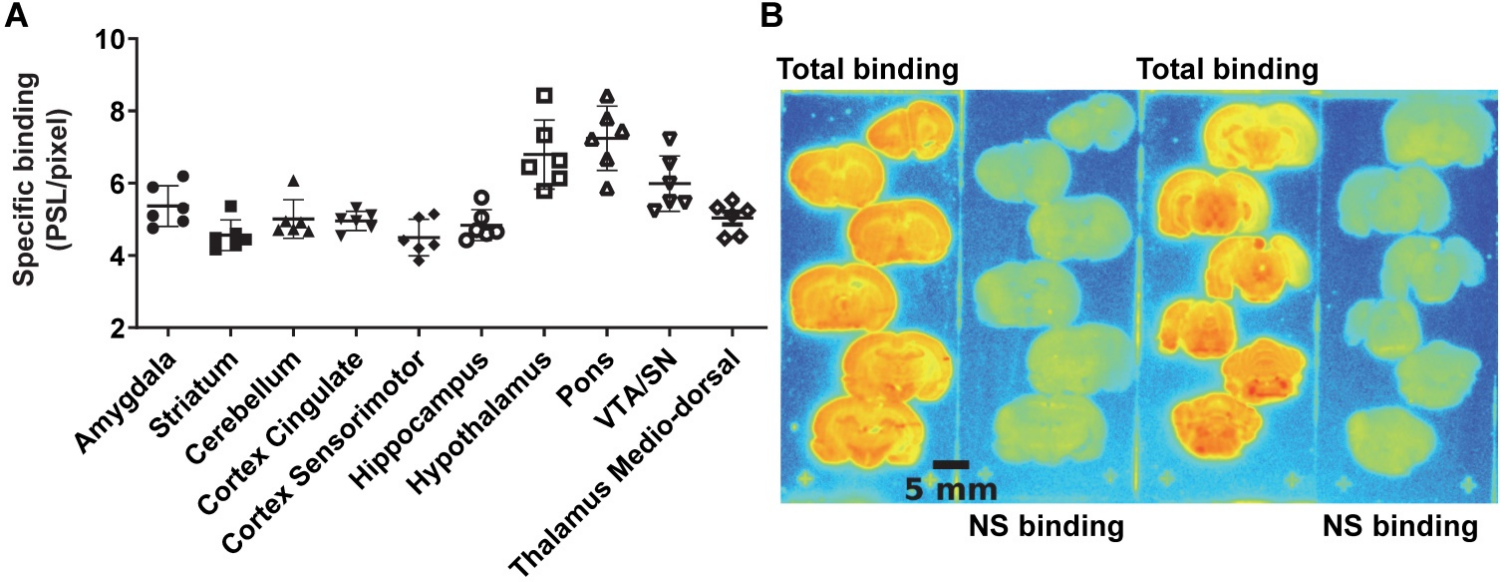
(**A**) quantification of ^18^F-IAM6067 specific binding as measured in rats by *in vitro* autoradiography in various brain regions. Data expressed as mean ± SD (n=6) of photostimulated luminescence (PSL) unit per pixel. VTA/SN: ventral tegmental area/*substantia nigra*. (**B**) Representative autoradiograms showing total and non-specific binding in different rat brain regions.

The average brain uptake of the radiotracer between 20 and 60 min post-injection in several brain regions is shown in Figures 2 & 3. Pharmacokinetic time-activity curves at baseline and after pre-saturation with BD1047 in the rat thalamus medio-dorsal and amygdala highlighted the specificity of ^18^F-IAM6067 bindings for the S1R (i.e. full blocking by BD1047, Figure 3A, C & E). ^18^F-IAM6067 brain uptake was rapid and attained a maximum level (3.3 and 5.5 SUV in amygdala and thalamus medio-dorsal respectively) at 2.25 min post-injection and then progressively and slowly washed out up to 60min. At baseline, the lowest average uptake of ^18^F-IAM6067 was detected in the caudate/putamen (2.3 ± 0.1 SUV), in the cingulate cortex (2.3 ± 0.2 SUV) and the temporal cortex (2.5 ± 0.2 SUV) (Figure 2A). The highest uptakes were found in the *substantia nigra* (3.8 ± 0.7), thalamus medio-dorsal (3.9 ± 0.4 SUV), mesencephalic region (4.7 ± 0.3 SUV) and pons-raphe (6.4 ± 0.8 SUV) (Figure 2A). Comparisons of ^18^F-IAM6067 uptake between all ROIs are shown Figure 2B. Those results were confirmed by ^18^F-IAM6067 autoradiography in rat brain regions which showed similar anatomical distribution of ^18^F-IAM6067 binding as the PET imaging (Figure 4). The highest specific binding was observed in the pons (7.24 ± 0.89 PSL/pixel) and the hypothalamus (6.79 ± 0.95 PSL/pixel) and the lowest specific binding in the striatum (4.56 ± 0.42 PSL/pixel) and the cingulate (4.96 ± 0.26 PSL/pixel) and somatosensory cortices (4.49 ± 0.51 PSL/pixel) (Figure 4).

Pre-saturation with SB206553 (5HT_2B/C_ antagonist, blue bars, Figure 3C & E) or CM398 (S2R, Figure 3B, D & E) did not reveal any significant difference with baseline uptake. Pre-saturation studies with haloperidol (D2/σ receptor antagonist; orange bars) or BD1047 (S1R antagonist; red bars) showed a significant decrease (between 79-90%) of ^18^F-IAM6067 uptake in all areas of the brain (Figure 3B, C & E) which abolished all differences between ROIs.

### Excitotoxic lesion model in rats

**Figure 5:**
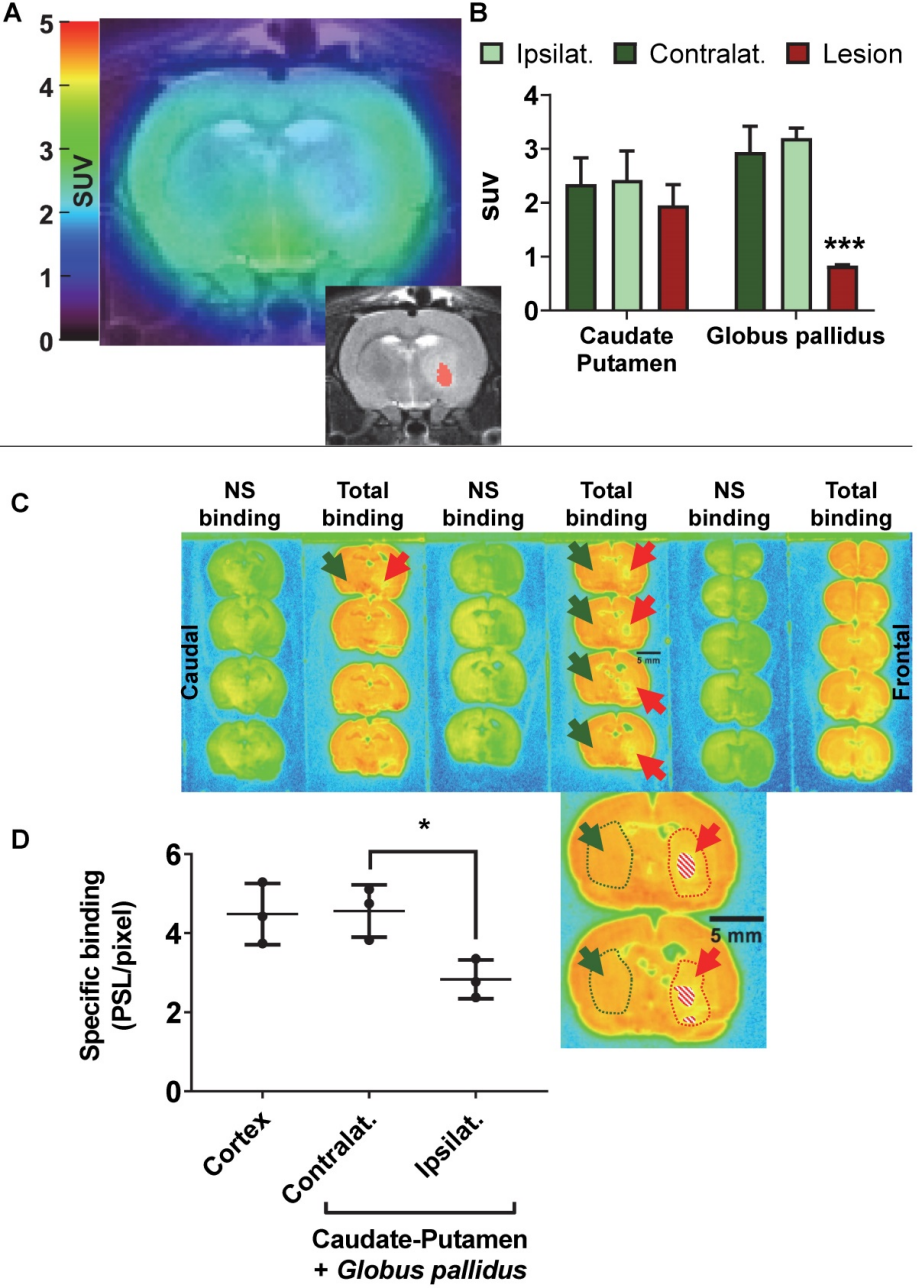
(**A**) representative ^18^F-IAM6067 PET-MR fused image and T2-MR image (insert, with the lesion in the *globus pallidus* as delineated on the T2 image in pink) 24h after intra-striatal injection of AMPA (7.5 nmol). (**B**) Quantification of the ^18^F-IAM6067 uptake in the ipsilateral (healthy), contralateral and lesioned Caudate-Putamen and Globus pallidus. (**C**) Representative autoradiograms of the same rat brain than in (**A**) showing the total and non-specific binding of ^18^F-IAM6067. The location of the lesion (minus piece of missing tissue indicated by the dashed pattern) is delineated by red dotted line and arrows while the contralateral side is delineated by green dotted line and arrows. (**D**) Quantification of the specific binding as quantified by autoradiography in the cortex and contralateral and ipsilateral caudate-putamen+*globus pallidus*. Data are expressed as mean ± SD (n=3). PET data were analysed with a 2 way ANOVA (brain structures & side, ipsi- *vs* contralateral) followed by a Sidak's post-hoc test. Contra- and ipsilateral sides for the autoradiography were compared with a paired *t*-test. * And *** indicates p<0.05 and p<0.001 respectively.

**Figure 6:**
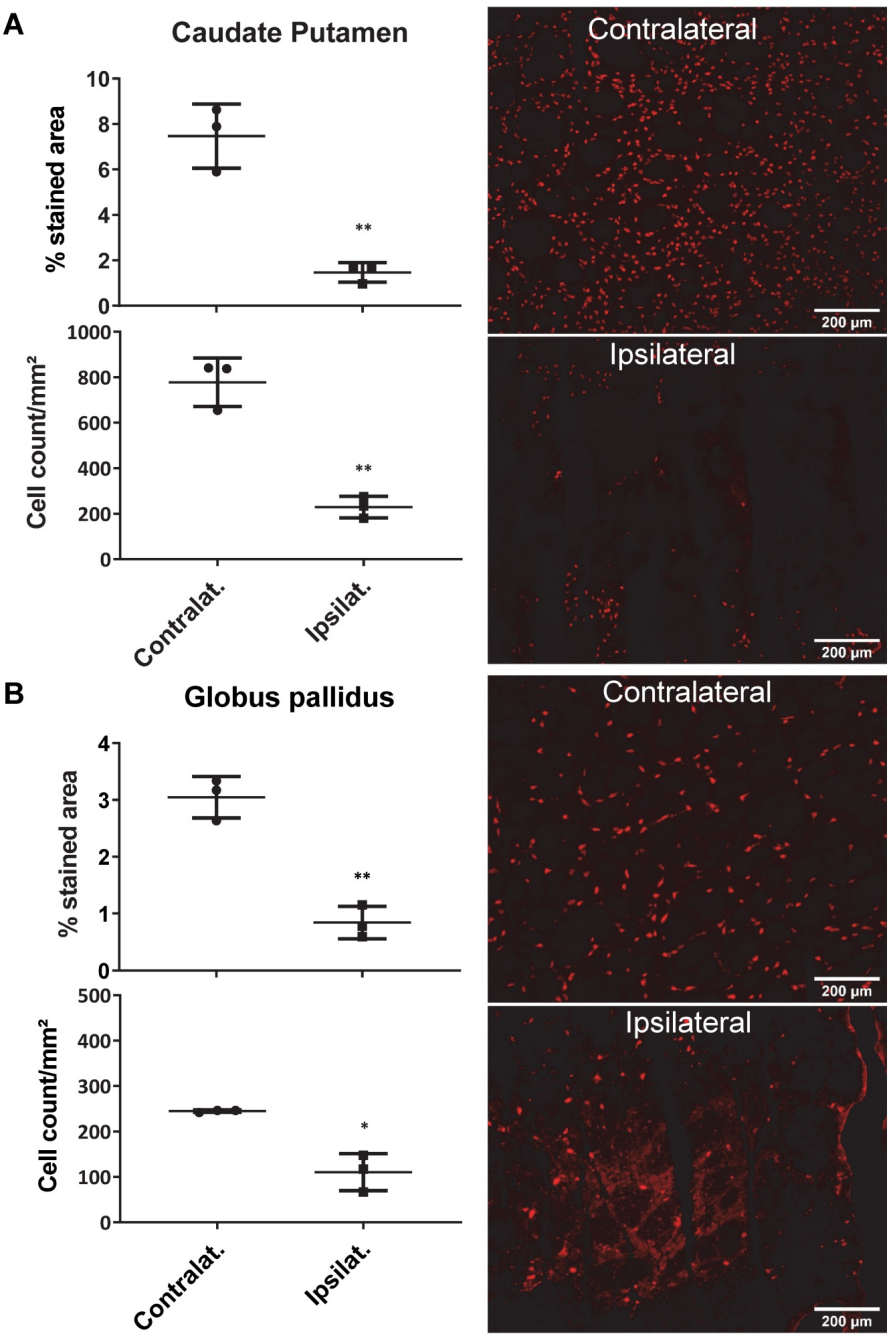
Quantification of the neuronal density (as percentage of stained area and cells/mm²) (left panel) and representative images of the NeuN immunostaining in the ipsi- and contralateral side of the caudate-putamen (**A**) and *globus pallidus* (**B**) 24h after intrastriatal AMPA injection in rats. Data are shown as mean ± SD. Data were analysed using paired t-tests. * And ** indicate p<0.05 and p<0.01 respectively.

AMPA injection in the striatum resulted in a significant neuronal loss 24h post-injection characterised by oedema visible on the T2 MRI (Figure 5A) and as quantified by NeuN staining (Figure 6). ^18^F-IAM6067 PET imaging revealed only a non-significant trend to decrease in ^18^F-IAM6067 uptake in the caudate-putamen (-16 ± 11%, Figure 5B) despite a significant loss of neurons (-81 ± 3% in % stained area and -71 ± 3% in cell number, Figure 6A), ^18^F-IAM6067 uptake was however significantly reduced in the *globus pallidus* (-69 ± 5%, Figure 5B) matching the significant loss of neurons detected by NeuN immunohistochemistry in this ROI (-71 ± 8% in % stained area and -56 ± 17% in cell number, Figure 6B). Comparing all the ROIs in control animals (no lesion) with AMPA-lesioned rats revealed a significant increase in ^18^F-IAM6067 uptake in both ipsi- and contralateral *Substantia nigra* and pons-raphe (Supplementary Figure 1A and B) although no changes in NeuN immunostaining could be detected in those brain regions (Supplementary Figure 1C); ^18^F-IAM6067 uptake was not affected in any other brain areas (Supplementary Figure 1A and B).

The post-mortem autoradiographic analysis of the ^18^F-IAM6067 binding in the ipsi- *vs* contralateral striatum (caudate-putamen + *globus pallidus*) of the same animals revealed a significant decrease (-38 ± 8%, Figure 5C & D).

### 6-OHDA Parkinson's disease model in mice

**Figure 7:**
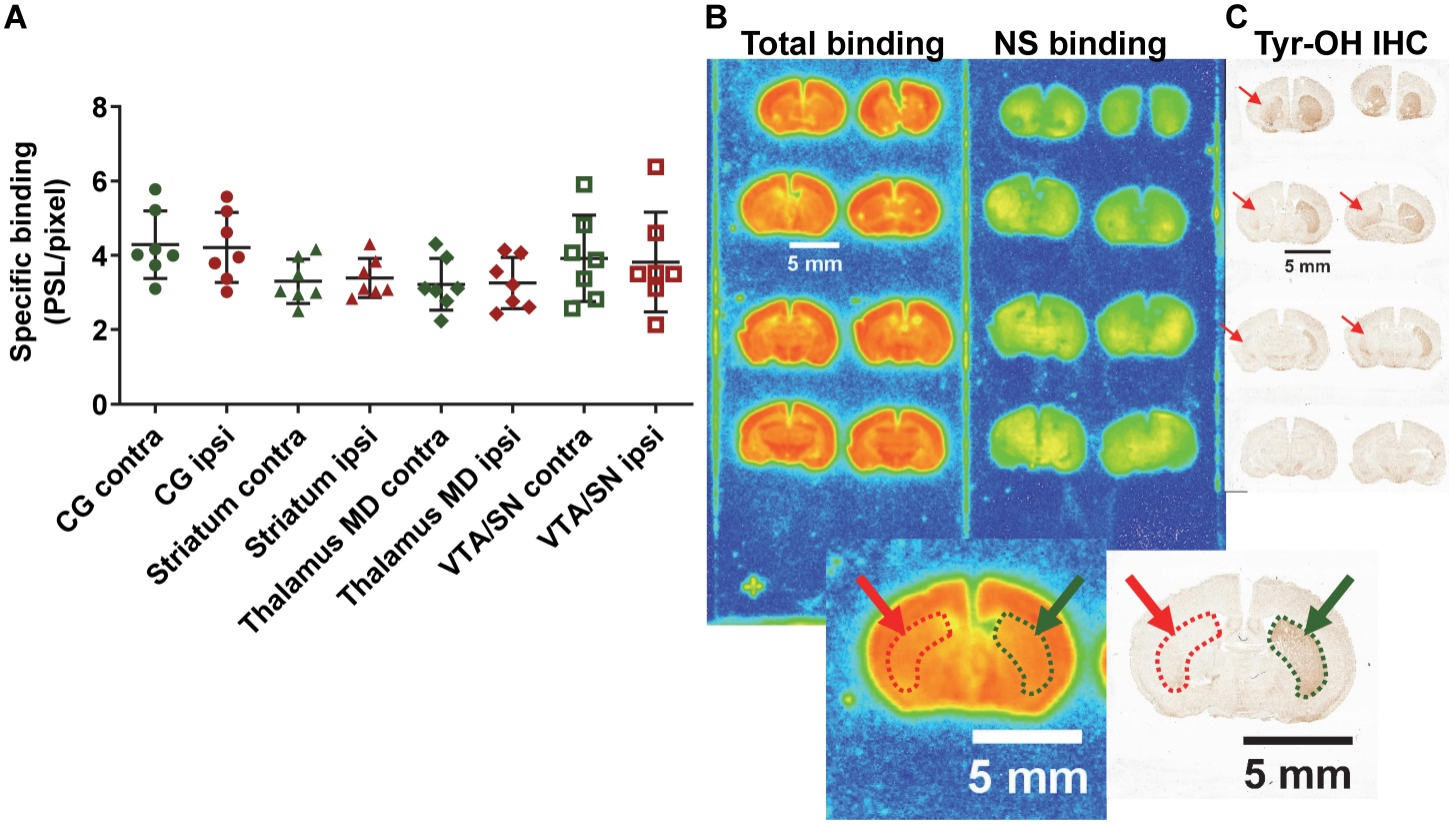
(**A**) Quantification by autoradiography of the ^18^F-IAM6067 specific binding in various contralateral and ipsilateral brain regions of the 6-OHDA Parkinson's disease mouse model. Data are expressed as mean ± SD (n=7). (**B**) Representative autoradiograms of a mouse brain showing the total and non-specific binding of ^18^F-IAM6067. (**C**) Representative immunohistological staining against tyrosine hydroxylase (Tyr-OH IHC) showing the significant loss of dopaminergic neurons in the ipsilateral striatum. The location of the lesion is delineated by red dotted line and arrows while the contralateral side is delineated by green dotted line and arrows on both the autoradiograms and immunostaining. NS: non-specific; CG: cingulate cortex; MD: medio-dorsal; VTA/SN: ventral tegmental area/*substantia nigra*. Data were analysed using a 2 way ANOVA (brain structures & side, ipsi- *vs* contralateral), there was no significant differences between ipsi- and contralateral side and no significant interaction between ROI and side.

In the PD mouse model, the 2 way ANOVA returned an effect of ROI (p=0.003) only. No differences between ipsilateral and contralateral sides or interaction ROI × side were identified (p=0.84 and p=0.65 respectively) (ipsi- *vs* contralateral; cingulate cortex: 4.21 ± 0.87 *vs* 4.29 ± 0.84 PSL/pixel, striatum: 3.39 ± 0.49 *vs* 3.30 ± 0.55 PSL/pixel, thalamus medio-dorsal: 3.25 ± 0.64 *vs* 3.22 ± 0.65 PSL/pixel and ventral tegmental area/*substantia nigra*: 3.82 ± 1.25 *vs* 3.92 ± 1.08 PSL/pixel) despite evidence of a clear lesion of the dopaminergic neurons as detected by tyrosine hydroxylase immunohistochemistry (Figure 7).

### Autoradiographic study of brains from Alzheimer's disease patients

**Figure 8:**
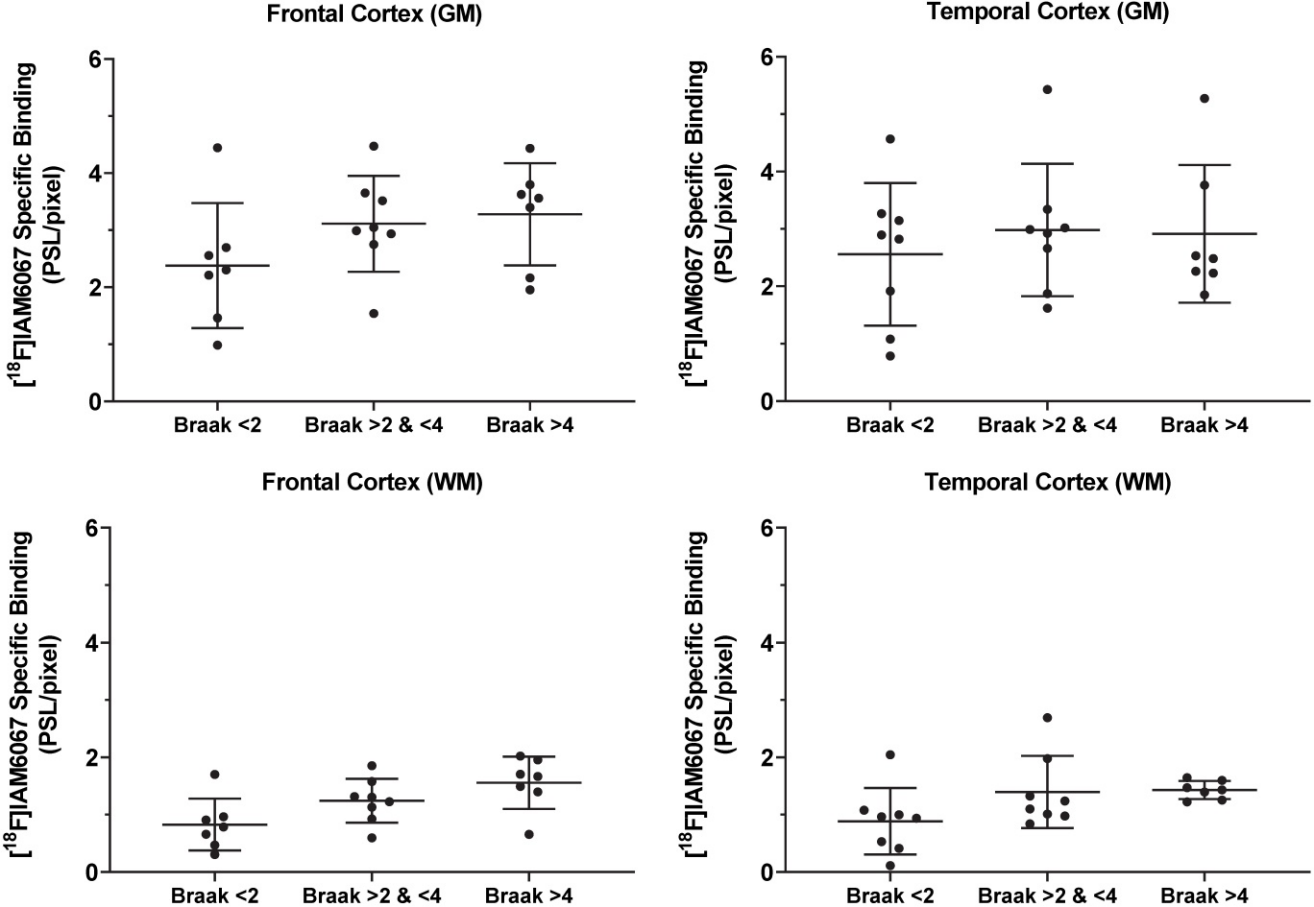
Quantification by autoradiography of the ^18^F-IAM6067 specific binding in the grey (top panel) and white matter (bottom panel) of control subjects (Braak ≤2, n=7-8), subclinical AD (Braak >2 & ≤4, n=8) and AD patients (Braak >4, n=7) in frontal cortex (left panel) and in temporal cortex (right panel). Data are expressed as mean ± SD. Data were analysed by mixed-model fitting (brain structures as repeated factor). As this analysis only returned an effect for the brain structures factor (p<0.001) and not for the Braak stage (p=0.095) or interaction brain structure × Braak stage (p=0.91), a Sidak's post-hoc test comparing brain regions only was performed, for both brain structure and all Braak stage, specific binding in white matter was significantly lower than in grey matter.

The specific binding of^ 18^F-IAM6067 was also investigated by autoradiography in the grey matter of controls (Braak stage ≤2), subclinical AD (Braak stage >2 & ≤4) and AD (Braak stage >4) subjects in the frontal and in the temporal cortex. Only a significant difference between grey and white matter could be detected, white matter being significantly lower than grey matter (Figure 8). No differences between Braak stage or interaction brain structures × Braak stage were detected (p=0.095 and p=0.91, respectively) (2.37 ± 1.09 *vs* 3.11 ± 0.84 *vs* 3.27 ± 0.90 PSL/pixel, for controls, subclinical AD and AD groups, respectively, in the frontal grey matter and 2.56 ± 1.24 *vs* 2.98 ± 1.15 *vs* 2.91 ± 1.20 PSL/pixel, for controls, subclinical AD and AD groups, respectively, in the temporal grey matter) (Figure 8).

## Discussion

### Brain uptake, distribution and selectivity of ^18^F-IAM6067

Here we investigated the S1R PET tracer ^18^F-IAM6067 and demonstrated that brain distribution was in good agreement with the known distribution of S1R in the rat brain 54-56. Despite rapid metabolism in plasma, ^18^F-IAM6067 brain uptake was fast and allowed for accurate and specific quantification of S1R by PET imaging. Using two different animal models of neurodegeneration and human brain samples, our results suggest that the expression levels of S1R might not be directly related to neuronal loss and might be subject to more complex regulation.

The S1R-system regulates most of the major neurotransmission systems such as cholinergic, noradrenergic and dopaminergic systems 10 in the CNS and as such S1R has emerged as a potential biomarker of neuronal function. For example, Mishina *et al.* have reported a decrease in S1R density in PD patients 17 in the anterior putamen and reduction in cortical and cerebellar S1R density in brains of AD patients 18 and others have reported decreases in S1R density in *post-mortem* tissue of AD patients 19, 20. Therefore, the evaluation of suitable (i.e. high selectivity notably *vs* S2R and other targets) S1R radiotracers is still required to investigate S1R density non-invasively *in vivo*, notably because many S1R tracers developed in the past displayed low affinity for S1R, unfavourable brain penetration 57 and/or insufficient σ1/σ2 selectivity. ^18^F-FTC-146 26 and ^18^F-fluspidine 27 are however the two noteworthy exceptions of previously reported ^18^F-labelled S1R tracers with high S1R affinity and σ1/σ2 selectivity. ^18^F-FTC-146 has been used in a preclinical model 58 and in a clinical case of peripheral injury 59 although it was also found to have slow brain pharmacokinetic for its use in human 30, 31. Conversely, (S)-^18^F-fluspidine seems to have suitable pharmacokinetics and metabolism in human 60, 61 and is now being used in clinical studies 62, 63. ^18^F-IAM6067 had already been shown to have sufficient selectivity against S2R (K_i σ2/σ1_ = 187) and other targets (see supplementary data in 29 in which ^18^F-IAM6067 is compound #30 page S3). We present here the evaluation of ^18^F-IAM6067 as a S1R specific PET tracer and confirmed its σ1/σ2 suitable selectivity *in vivo* (Figure 3D). Metabolite analysis revealed a rapid metabolism of the parent fraction of ^18^F-IAM6067 in rat plasma (approximatively 10% at 10 and 20 min, and not detectable at 60 min post-injection) (Table 2). Conversely, in brain most of the radioactivity detected was due to the parent compound (≥91% of the intact tracer at 10, 20 and 60 min post-injection) and only a small amount of metabolites detected in plasma could be detected in brain. While we cannot totally exclude that the metabolites were able to cross the blood-brain barrier (BBB), the small amount detected (8.0 ± 6.2% of the total activity 60 min post-injection) as well as the low HPLC retention time (1.4 and 1.8min), suggesting a high hydrophilicity, support the hypothesis that the metabolites detected in brain are from blood left in the samples rather than from crossing the blood brain barrier. This would therefore support the notion that the ^18^F-IAM6067 brain signal detected in our experiments was most likely caused by intact radiotracer in the brain. Pre-saturation studies with haloperidol (non-selective Dopamine D2/σ1 receptors antagonist) or BD1047 (S1R specific antagonist) resulted in 79-90% blocking of ^18^F-IAM6067 uptake (see Figure 3A, C & E) and abolished all ROI differences, hence indicating presence of specific binding in all brain areas. To further verify the selectivity of ^18^F-IAM6067 *vs* S2R, we performed repeated scans with and without pre-saturation with the highly selective S2R ligand CM398 (K_i σ1/σ2_ = 331) 36. Injection of cold CM398 did not result in a significant reduction of ^18^F-IAM6067 uptake (Figure 3B, D & E). We also assessed specificity towards 5HT_2B/C_ receptors as Moussa *et al.* 29 reported a K_i_ ratio (5HT_2B_/S1R) of only 37, when compared with the σ2/σ1 ratio of 187. Our pre-saturation experiment using 5HT_2B/C_ antagonist SB206553 showed no significant difference in brain uptake in any of the brain ROIs studied (Figure 3C & E), hence supporting the selectivity of ^18^F-IAM6067 towards S1R *vs* 5HT_2B/C_ receptors. Overall, the results of the pre-saturation studies and the presence of specific binding throughout the brain are in agreement with the previous reports 10, 28. However, the presence of specific binding in all ROIs prevents the use of any brain region as a reference tissue for modelling purpose. From an anatomical point of view, our autoradiography study in rats was in good agreement with our *in vivo* PET study. Overall, these results validate the fact that ^18^F-IAM6067 is a suitable specific and selective S1R radiotracer, although the slow pharmacokinetic of ^18^F-IAM6067 in primate 28 may limit its usefulness in clinical studies.

### Evaluation of ^18^F-IAM6067 and S1R as biomarker of neuronal loss

The second objective of this study was to assess S1R as a biomarker of neuronal function in focal neurodegenerative disease models. First, we used an acute model of neurodegeneration induced by AMPA excitotoxicity in the striatum. As previously reported by us and others 64-66, intrastriatal injection of AMPA induces robust neuronal death within 24h followed by a neuroinflammatiory response. Here we obtained a consistent 70-80% loss of neurons in the striatum as demonstrated by NeuN immunostaining (Figure 6), but despite this the decrease in ^18^F-IAM6067 uptake as quantified by PET was inconsistent, with a significant decrease in the *globus pallidus* (Figure 5B) but only a trend to a decrease in the caudate-putamen; although the autoradiography analysis revealed a more consistent -38% decrease in specific binding in both the caudate-putamen and *globus pallidu*s. The inconsistency between PET imaging, autoradiography and immunohistochemistry could be due to partial volume effect for the PET imaging, the lesioned areas (caudate putamen and *globus pallidus*) are amongst the ROIs with the lowest level of S1R and are surrounded by other brain region in which the ^18^F-IAM6067 uptake is higher, consequently spill over in the lesion might explain the lack of amplitude in the decrease of PET signal when compared to autoradiography. The presence of focal lesion is also a different paradigm than assessed by Ishiwata *et al.* 67 and Ramakrishnan *et al.* 68 which studied the effect of aging on S1R binding. Firstly, these two reports investigated the overall effect of aging on the brain levels of S1R rather than disease associated neurodegeneration. Secondly, it is noteworthy that these two publications report contradictory results; Ishiwata *et al.* 67 reported a significant increase (×4.57 fold) in B_max_ accompanied by a significant decrease in affinity (increased K_d_, ×3.75 fold), resulting in an overall slight increase (×1.2 fold) in S1R binding potential (BP_ND_) whereas Ramakrishnan *et al.* 68 reported a significant decrease (approximately 2 fold) in S1R BP_ND_. Furthermore, two different strain of rats were used in these 2 studies: Fisher-344 67 and Wistar Hannover rats 68, so possible differences between strains cannot be ruled out. Moreover, the results of Ishiwata *et al.* 67 demonstrating a modulation of both B_max_ and K_d_ suggests a fine functional modulation of S1R rather than only a direct relation with neuronal loss. Interestingly, our observation of an increase in S1R in the *substantia nigra* (Supplementary Figure 1A & B) supports this theory of a fine modulation of S1R in response to neuronal stress or loss. So far, the meaning of such modulation of S1R in term of neuronal functionality remains to be elucidated. Second, we used 6-OHDA-lesioned mice, one of the most accepted animal models for PD 69. In this model, we were not able to find any difference in ^18^F-IAM6067 specific binding between the ipsilateral and contralateral hemispheres in the cingulate cortex, striatum, thalamus medio-dorsal and VTA/SN, despite clear loss of tyrosine hydroxylase positive neurons in the striatum (Figure 7), showing that the density or expression of S1R was not sensitive to the loss of these neurons. This result is in contradiction with the results by Mishina *et al.* 17 who investigated S1R density in 6 PD patients *vs* 6 controls by ^11^C-SA4503 PET. They demonstrated a decrease in S1R binding potential (BP) but only in the most affected anterior putamen (as assessed by ^11^C-CFT for dopamine transporter and ^11^C-raclopride for D2 receptor PET imaging) but interestingly did not detect differences between controls and PD patients when measuring ^11^C-SA4503 in the anterior putamen bilaterally 17. They suggested that this result supported the use of S1R BP as marker of loss of presynaptic dopaminergic neurons in PD 17. One must consider that the 6-OHDA model do not reproduce all the hallmarks of the human disease 70 and that Mishina *et al.* performed an *in vivo* PET imaging study whereas we used *ex vivo* autoradiography in mice, so differences in techniques and human *vs* animal model may have contributed to the discrepancy between the two studies. Finally, the studies cited above used ^11^C-SA4503 while we used ^18^F-IAM6067, and differences in binding sites between these ligands cannot be ruled out to explain potential differences between our results and those reported in these studies.

In order to further investigate whether S1R density is a reliable and sensitive biomarker of neuronal loss/dysfunction, we also used *post-mortem* brain sections from AD patients. Again, we showed no difference in ^18^F-IAM6067 specific binding in the frontal and temporal cortices between control subjects, patients with mild to moderate AD-type pathology and patients with established AD. Our data are in disagreement with previous data from the literature looking at AD patients. Indeed, the binding potential of ^11^C-SA4503, used in a 90min PET study, was significantly reduced in the frontal, temporal and occipital cortex as well as in the cerebellum and the thalamus in AD patients *vs* controls 18. Furthermore, Hedskog *et al.* 20 found a decrease of S1R expression in *post-mortem* AD cortical tissue by Western blot compared to controls. Additionally, an autoradiography study revealed a decrease (26%) in ^3^H-DTG binding in the hippocampus of AD patients *vs* controls 19. Conversely, Hedskog *et al.* 20 also showed that S1R was up-regulated in a mouse model of AD 20 while σ2 receptor density was decreased in a mouse model of AD in cortical regions and the striatum of female, but not male, mice 71. Again, differences between animal models and human disease could have contributed to the discrepancies between studies. However, there is also a discrepancy between our autoradiography study and the one by Jansen *et al.* 19, but it must be noted that ^3^H-DTG has a K_i_ *vs* non-selective S1R ligand haloperidol and opioid receptor ligand pentazocine of only 5 and 42nM respectively 72 so is not as specific for S1R as ^18^F-IAM6067. This poor selectivity might have led to the observation of decreases in binding sites encompassing S1R as well as other binding sites such as opioidergic receptors, which we have demonstrated to be very sensitive to neuronal loss in stroke 73, 74. Finally, there are other points to take into consideration when comparing our results with those previously published. Firstly, our control group pools together 'true' control cases (i.e. Braak 0) with Braak stage ≤2, this may have biased the value in our control group, although it is unlikely since these patients had no history of neurological disease and no pathological evidence of AD or other degenerative brain disease. Moreover, we know from neuropathological analysis that the brain of patients in the moderate and severe AD had far more degeneration and advanced neuropathology than those in the control group 75. Secondly, the number of cases used in the study by Hedskog *et al.* 20 was lower (3 and 3) than in our study where we used 23 cases split in 3 groups (n=7-8 per group), which would seem more robust. Thirdly, the cases of our study had a higher *post-mortem* delay (38.6 ± 12.9 hours for all patients) than those used by Hedskog *et al.* 20 (between 10 and 30 hours) or Jansen *et al.* 19 (14 ± 5 and 12 ± 6 hours for AD patients and controls, respectively), and while these were the cases with the shortest *post-mortem* delay we could access, this may have influenced the quality of the tissue and the levels of detectable S1R. Finally, age is the best known risk factor for AD 76, so it is important to know how S1R expression changes with age. Overall, it was shown by PET imaging in rats that S1R could be either slightly increased 67 or decreased 68 with age, while in AD S1R has been shown to increase in animal model 20 but conversely to decrease in human studies 18-20. Therefore, prediction of how S1R density might evolve in neurodegenerative disease such as AD, and the use of S1R as direct marker of neuronal loss, might not be as straight forward as it once seemed with age being a significant confounding factor.

While we cannot totally rule out a potential lack of sensitivity of ^18^F-IAM6067 in our experimental settings, the absence of decreases in S1R binding in the mouse PD model and AD brains, the increase in the *substantia nigra* in the AMPA model in rats (Supplementary Figure 1A & B), taken together with previous reports 17, 18, 20, 67, 68 suggest that S1R expression and/or binding characteristics are altered differently in different population of neurons or depending on the paradigms. In the absence of similar studies in animal models of neurodegeneration and human with tracers such as ^18^F-fluspidine and considering that S1R is a chaperone protein and not a neurotransmitter receptor, our results suggest that the interpretation of changes in S1R PET tracer uptake may be more difficult to interpret than initially thought as these may not purely reflect a neuronal loss or dysfunction but also more complex compensatory mechanisms.

## Conclusion

The present *in vivo* PET study in rats shows that ^18^F-IAM6067 is a suitable specific and selective S1R radiotracer in rodent. Considering the relatively slow pharmacokinetics in baboon shown by Moussa *et al.* 28, ^18^F-IAM6067 may not provide any advantage over the currently used (S)-^18^F-fluspidine for imaging S1R in human 62, 63. Our data in the rat AMPA, mouse PD models and in human brain tissue did not allow us to conclude positively on the potential use of S1R as biomarker for neuronal (dys)function in neurodegenerative conditions such as AD or PD. Reports about changes in S1R density with age and disease are conflicting so far, and with the emergence of S1R as a potential therapeutic target in neurodegenerative diseases and in tumours, further elucidation of the expression, function, density and role of S1R is of highest interest. Overall the previously published data and the present study warrant further investigations on the S1R-system in health and disease.

## Figures and Tables

**Table 1:** Clinical summary of autopsy cases used for the ^18^F-IAM6067 autoradiography experiments

Case no.	Gender	Age of death (years)	PM delay (hours)	Principal pathological diagnosis	Age of disease onset (years)	Braak stage
1	M	54	37	Normal brain	N/A	≤2
2	M	95	12	Age changes only	N/A	≤2
3*	F	72	41	Age changes only	N/A	≤2
4	F	81	41	Age changes only	N/A	≤2
5	F	82	46	Age changes only	N/A	≤2
6	F	89	41.5	Age changes only	N/A	≤2
7	F	87	24	Age changes only	N/A	≤2
8	F	92	37	Age changes only	N/A	≤2
						
9	M	82	40	Moderate AD	75	>2 & ≤4
10	F	93	41	Mild AD	N/A	>2 & ≤4
11	M	89	48	Mild AD	N/A	>2 & ≤4
12	F	92	25	Severe CAA	84	>2 & ≤4
13	M	91	33	Moderate AD	84	>2 & ≤4
14	M	86	26	AD	N/A	>2 & ≤4
15	F	97	25	AD	N/A	>2 & ≤4
16	F	84	48	AD	78	>2 & ≤4
						
17	M	62	50	AD	56	>4
18	M	73	36	AD	60	>4
19	F	85	24	AD	N/A	>4
20	F	91	45	AD	N/A	>4
21	F	82	46	AD	68	>4
22	F	71	64	AD	64	>4
24	F	81	25.5	AD	74	>4

PM: *post-mortem* delay; CAA: cerebral amyloid angiopathy; AD: Alzheimer's disease; N/A: not available/applicable. * Measurements from frontal cortex of case #3 were not included as the sections had been damaged during the experiments.

**Table 2:** Percentage of ^18^F-IAM6067 (parent fraction and metabolites) in rat brain and plasma at 1, 2, 5, 10, 20 and 60 min post-injection. R_t_ indicates the retention time of each fraction of the radiotracer. Data expressed as mean ± SD

	Metabolites	R_t_	Time (min)						
			1	2	5	10	20	60	
Brain	M1	1.4	ND	ND	ND	5.6±2.7	3.8±1.9	8.0±6.2	Percentage of ^18^F-IAM6067 and metabolites
	M2	1.8	ND	ND	ND	3.9±2.0	2.6±0.8	1.5±0.7	
	Parent	11.1	ND	ND	ND	91.3±3.7	94.1±2.8	91.3±6.0	
Plasma	M1	1.4	8.9±8.1	41.5±4.4	77.6±11.6	84.4±17.3	87.3±19.1	99.7±0.8	
	M2	1.8	7.0±0.0	0.0	0.0	24.4±5.8	24.1±20.0	2.1±0.0	
	Parent	10.9	88.8±12.1	58.3±4.1	22.4±11.6	10.1±8.6	9.8±4.2	0.0	

M1: metabolite 1; M2: metabolite 2; ND: not determined. R_t_ = retention time (min).
