# Evaluation of ^18^F-IAM6067 as a sigma-1 receptor PET tracer for neurodegeneration *in vivo* in rodents and in human tissue: Erratum

**DOI:** 10.7150/thno.76351

**Published:** 2022-07-08

**Authors:** François-Xavier Lepelletier, Matthias Vandesquille, Marie-Claude Asselin, Christian Prenant, Andrew C Robinson, David M A Mann, Michael Green, Elizabeth Barnett, Samuel D Banister, Marco Mottinelli, Christophe Mesangeau, Christopher R McCurdy, Inga B Fricke, Andreas H. Jacobs, Michael Kassiou, Hervé Boutin

**Affiliations:** 1 Faculty of Biology, Medicine and Health, School of Biological Sciences, Division of Neuroscience and Experimental Psychology, University of Manchester, Manchester, United Kingdom.; 2 Wolfson Molecular Imaging Centre, University of Manchester, Manchester, United Kingdom.; 3 Faculty of Biology, Medicine and Health, School of Health Sciences, Division of Informatics, Imaging and Data Sciences, University of Manchester, Manchester, United Kingdom.; 4 Salford Royal NHS Foundation Trust, Department of Clinical & Cognitive Neurosciences, Clinical Sciences Building, Salford, United Kingdom.; 5 School of Chemistry, The University of Sydney, Sydney, Australia.; 6 Department of Medicinal Chemistry, College of Pharmacy, University of Florida, Gainesville, FL 32610, USA.; 7 Department of BioMolecular Sciences, School of Pharmacy, University of Mississippi, University, MS 38677, USA.; 8 UF Translational Drug Development Core, University of Florida, Gainesville, FL 32610, USA.; 9 European Institute for Molecular Imaging (EIMI), Westfälische Wilhelms-Universität (WWU), Münster, Germany.; 10 Department of Geriatrics and Neurology, Johanniter Hospital, Bonn, Germany.

The authors regret that the original version of our paper unfortunately contained incorrect data in Figure 2B, where the vertical plot of the ROIs significantly different of the mesencephalic region (x-axis, right hand side of the graph) were incorrect and did not match the horizontal level of significant differences. The correct version of the Figure 2B is shown below.

The corrections made in this erratum do not affect the original conclusions. The authors apologize for any inconvenience that the errors may have caused.

## Figures and Tables

**Figure 2:**
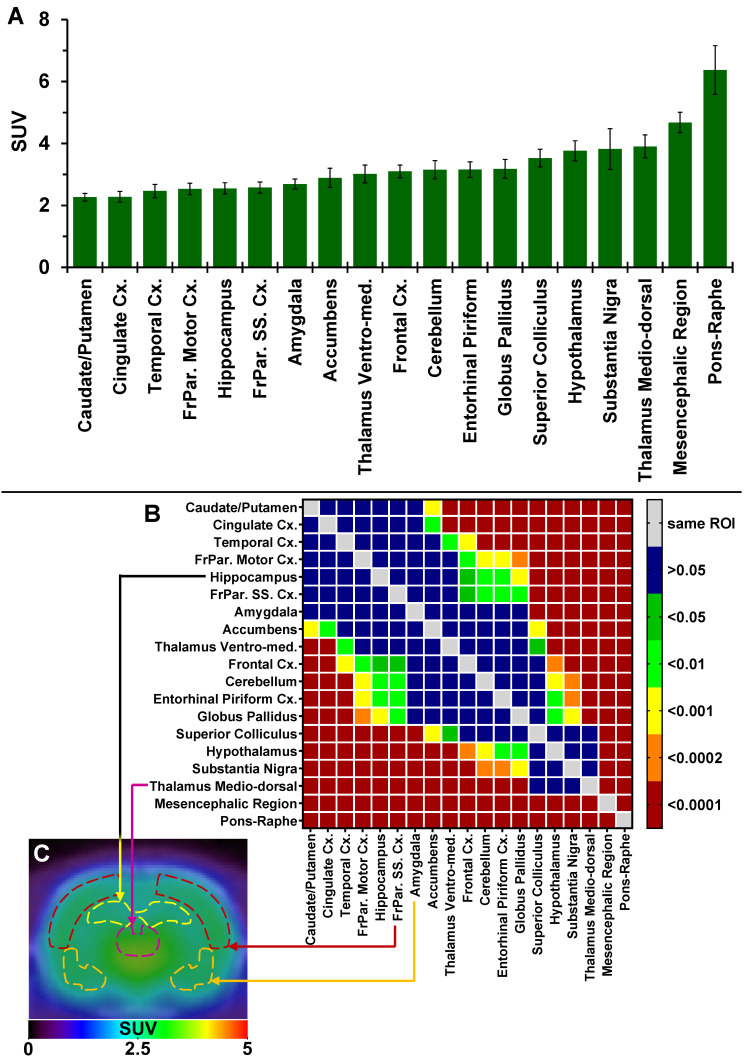
(**A**) Average uptake (from sum-image 20-60min post-injection) of ^18^F-IAM6067 in different brain regions in rats (n=8, data are expressed as SUV mean ± SD). (**B**) Heat map of the adjusted P values (Sidak's post-hoc test) showing all the comparisons between the various brain regions for ^18^F-IAM6067 uptake. Non-significant differences are shown in blue. (**C**) PET sum-image (20-60min) co-registered with CT showing ^18^F-IAM6067 uptake with hippocampus, thalamus medio-dorsal, frontoparietal somatosensory cortex and amygdala highlighted by dotted lines.

