# Supplementary material for: Evaluation of 18F-IAM6067 as a sigma-1 receptor PET tracer for neurodegeneration in vivo in rodents and in human tissue
Source: Theranostics. 2020 Jun 29;10(18):7938–55. doi: 10.7150/thno.47585 (PMC7381740; doi:10.7150/thno.47585)
Supplement: Supplementary file 1 — Supplementary figures. [file thnov10p7938s1.pdf]

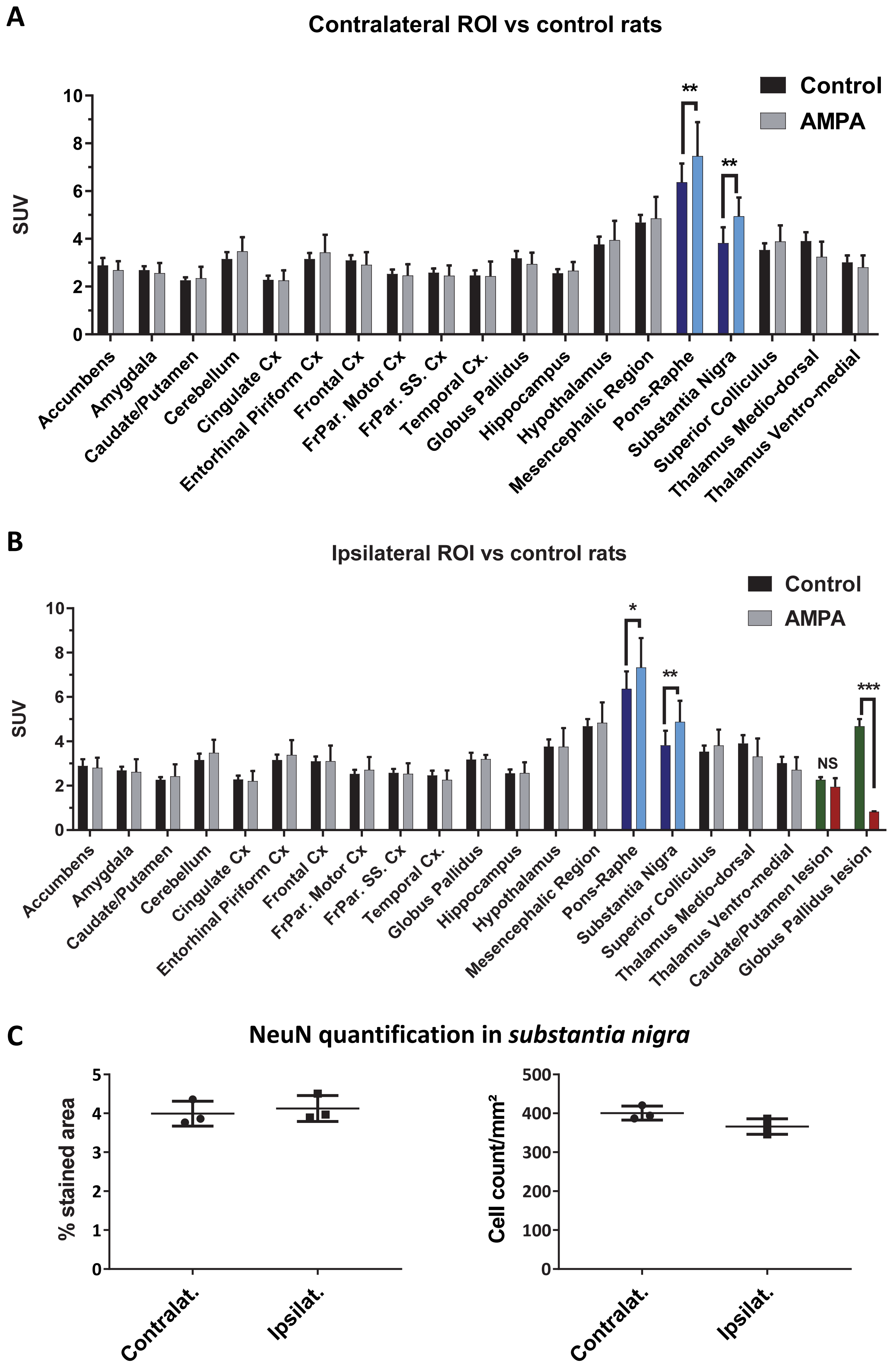
A
Contralateral ROI vs control rats
Control
AMPA
SUV
10
8
6
4
2
0
**
**
Accumbens
Amygdala
Caudate/Putamen
Cerebellum
Cingulate Cx
Entorhinal Piriform Cx
Frontal Cx
FrPar. Motor Cx
FrPar. SS. Cx
Temporal Cx.
Globus Pallidus
Hippocampus
Hypothalamus
Mesencephalic Region
Pons-Raphe
Substantia Nigra
Superior Colliculus
Thalamus Medio-dorsal
Thalamus Ventro-medial
B
Ipsilateral ROI vs control rats
Control
AMPA
SUV
*
**
***
NS
Caudate/Putamen lesion
Globus Pallidus lesion
C
NeuN quantification in *substantia nigra*
% stained area
Cell count/mm²
500
400
300
200
100
Contralat.
Ipsilat.

Supplementary Figure 1: Comparison of ^{18}F-IAM6067 brain uptake between control (naïve) (light bars) and AMPA injected rats (dark bars) in various brain regions. Note the significant increase in ^{18}F-IAM6067 brain uptake in both the ipsi- and contralateral *Substantia nigra* and pons-raphe (**A** & **B**, dark blue bars *vs* light blue bars) in AMPA injected animals *vs* controls and the significant decrease in the *Globus pallidus* (**B**, red *vs* green bars). (**C**) Quantification of the neuronal density (as % stained area and cells/mm^2) in the *Substantia nigra*. Data are expressed as mean ± SD. PET data were analysed with a 2 way-ANOVA followed by a Sidak's post-hoc test and immunostaining parameters were analysed using paired *t*-tests. ** And *** indicates $p<0.01$ and $p<0.001$ respectively. (FrPar. = frontoparietal, SS. = somatosensory, Cx = cortex).
